# Heavy Chain Deposition Disease in Monoclonal Gammopathy of Renal Significance: A Prodrome of Multiple Myeloma Case and Literature Review

**DOI:** 10.7759/cureus.85740

**Published:** 2025-06-10

**Authors:** Sahana Gnanasampanthan, Vasileios P Samelis, Candice Roufosse, Andreas Kousios

**Affiliations:** 1 Renal Medicine, Royal London Hospital, Barts Health NHS Trust, London, GBR; 2 Medicine, School of Medicine, European University of Cyprus, Nicosia, CYP; 3 Department of Immunology and Inflammation, Faculty of Medicine, Imperial College London, London, GBR; 4 Renal Medicine, School of Medicine, European University of Cyprus, Nicosia, CYP

**Keywords:** diagnosis of multiple myeloma, heavy proteinuria, kidney disease, monoclonal gammopathy of renal significance, monoclonal immunoglobulin deposition disease

## Abstract

Monoclonal immunoglobulin deposition disease (MIDD) is a complication of plasma cell dyscrasias, resulting in abnormal immunoglobulin deposition along basement membranes. We describe a case of a 60-year-old male with a complex hospital admission, presenting with critical illness accompanied by acute kidney injury, nephrotic syndrome and moderately elevated serum free light chain (SFLC) ratio, on a background of well-controlled diabetes, hypertension and chronic kidney disease. There was no clear aetiology for his presentation following preliminary examination and investigations, which led to a biopsy diagnosis of heavy chain deposition disease (HCDD) in the context of monoclonal gammopathy of renal significance (MGRS). We explore the importance of understanding the disease course to allow timely biopsy diagnosis and treatment initiation. Our patient required very close follow-up and a wide multi-disciplinary approach, including haematologists, nephrologists and histopathologists, to guide management in a disease for which therapeutic strategies are poorly defined due to limited clinical trial data.

## Introduction

Plasma cell dyscrasias may affect the kidney in many ways, presenting with a wide range of distinct renal histopathological patterns. Certain monoclonal immunoglobulins produced by the pathogenic plasma cell clone have nephrotoxic physiochemical properties and cause renal disease even if the haematological burden is low. The term monoclonal gammopathy of renal significance (MGRS) was introduced to describe these entities, where the criteria for haematological malignancy are not met, but the nephrotoxic monoclonal immunoglobulins cause progressive chronic kidney disease (CKD) that can lead to end stage kidney disease (ESKD) [1]. MGRS is different from the common monoclonal gammopathy of undetermined significance (MGUS), a condition which is considered benign, with very low risk of progression and does not warrant haematological treatment. However, in some cases it may progress to MGRS or multiple myeloma. The prevalence of MGRS is estimated to be less than 4-5% in native renal biopsies. MIDD is a rare renal histopathological diagnosis that can be seen in MGRS or multiple myeloma.

## Case presentation

A 60-year-old gentleman with well-controlled type 2 diabetes and hypertension was initially admitted to intensive care for chest sepsis requiring intubation. After a prolonged intensive care unit stay, he improved clinically but remained with oedema anasarca. His echocardiogram and cardiac MRI, showed left ventricular hypertrophy with preserved left ventricular ejection fraction (there was no intraventricular septum thickness).

Further investigations for his oedema, revealed nephrotic syndrome (proteinuria 5 gr/day, serum albumin 2.5g/dl). Autoimmune, complement levels and viral screen (including anti-PLA2r) was unremarkable. Serum free light chain (SFLC) kappa:lambda (k:l) ratio was mildly raised (2.96), just above reference range adjusted for renal function. Serum protein electrophoresis (SPEP), immunofixation and urine Bence Jones protein were negative.

**Figure 1:**
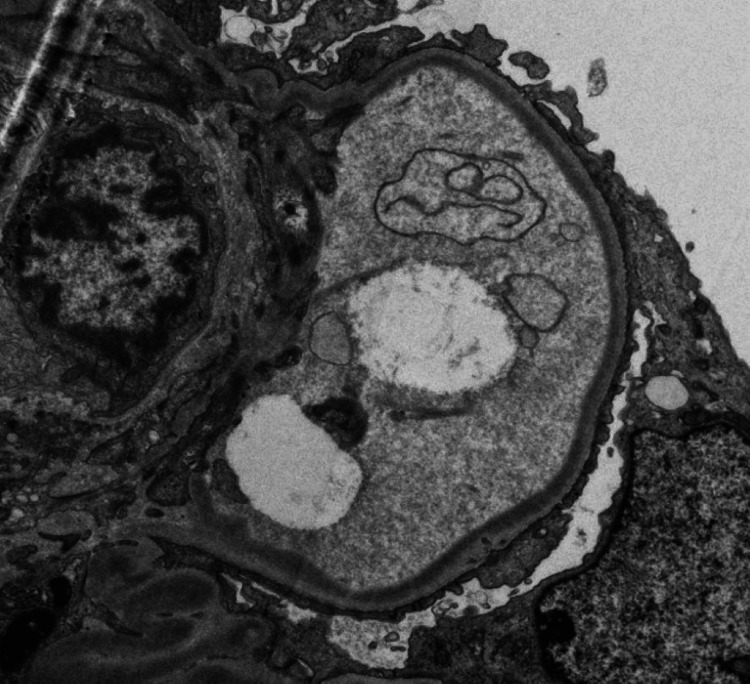
Electron Microscopy Electron microscopy showing the presence of linear, powdery, electron dense deposits along the endothelial aspect.

**Figure 2:**
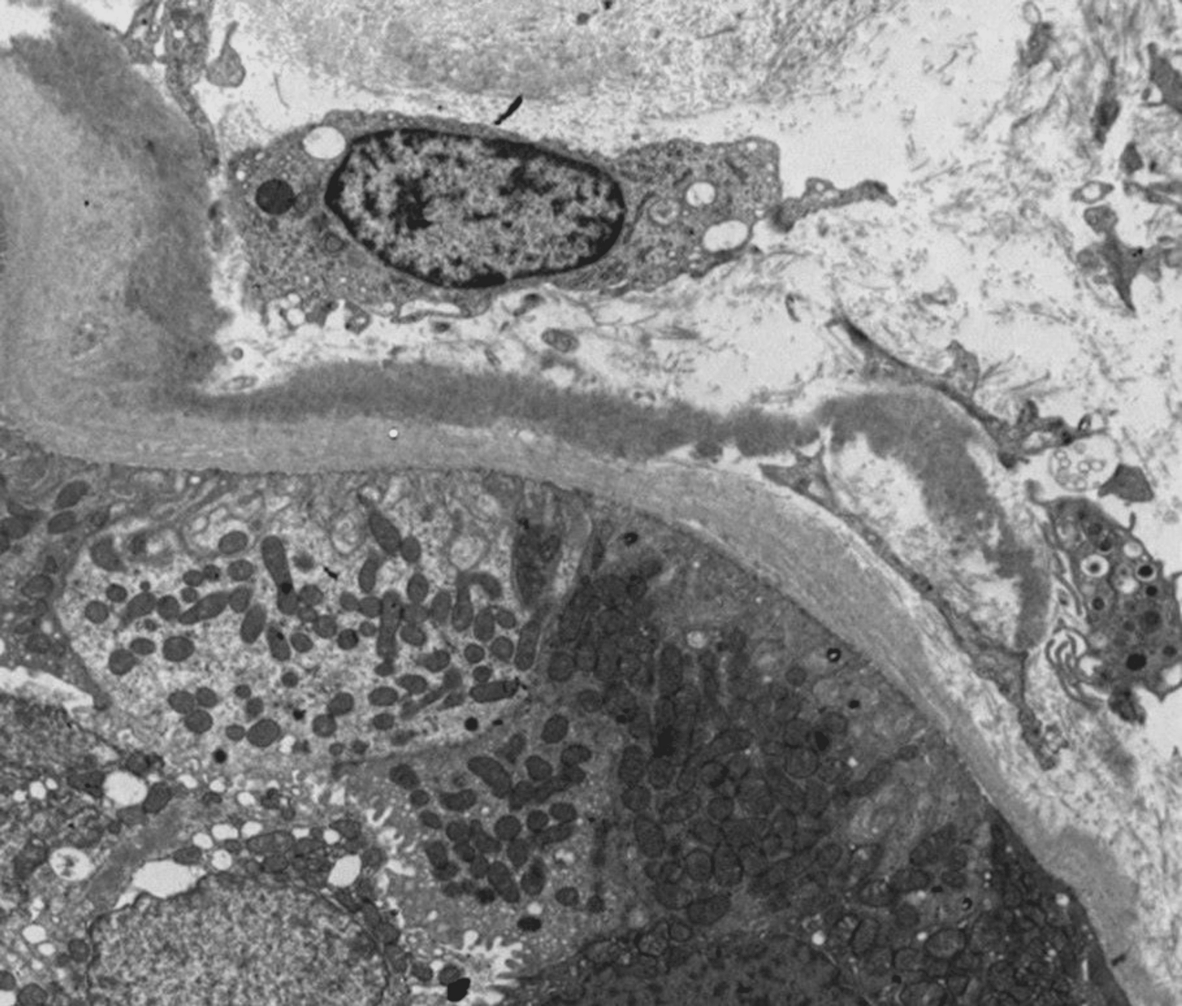
Electron Microscopy Electron microscopy showing the presence of linear, powdery, electron dense deposits  in the tubular basement membrane (TBM).

**Figure 3:**
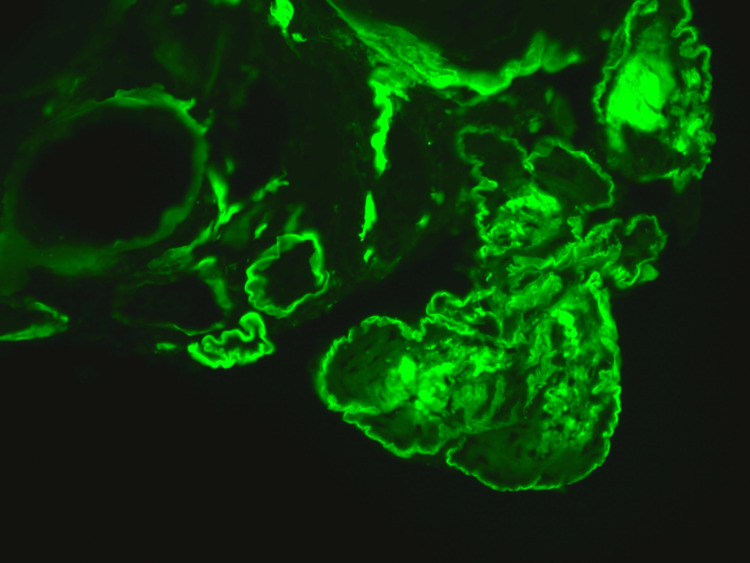
Immunofluorescence Direct immunofluorescence of the same glomerulus, showing strong positivity for immunoglobulin G (IgG).

**Figure 4:**
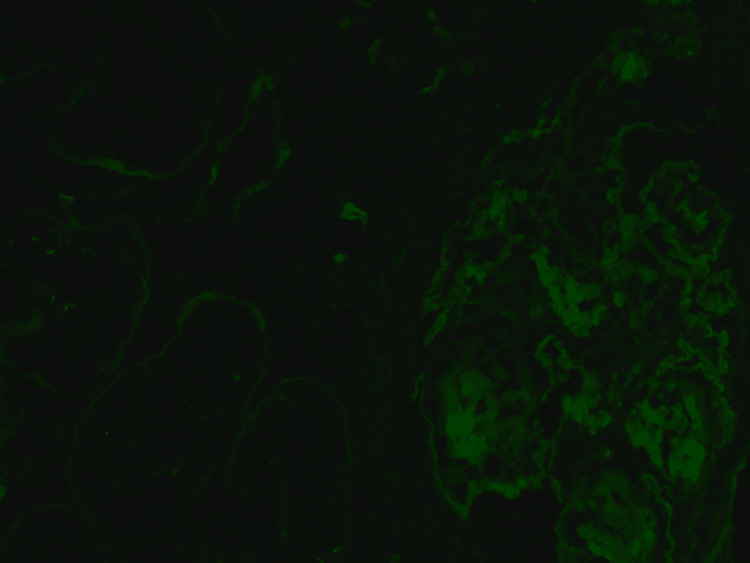
Immunofluorescence - kappa light chains Direct immunofluorescence of the same glomerulus and without positivity for kappa light chains.

**Figure 5:**
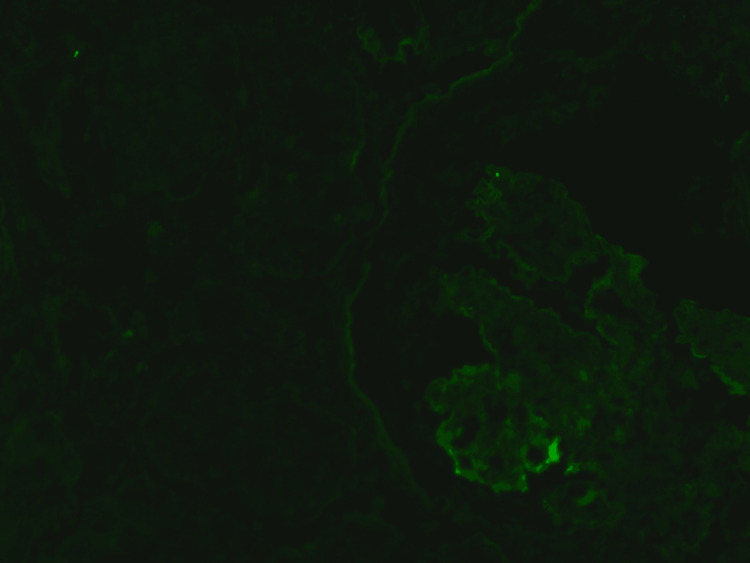
Immunofluorescence - lambda light chains Direct immunofluorescence of the same glomerulus without positivity for lambda light chains.

A kidney biopsy was performed, showing acute tubular injury. All glomeruli were abnormal, lobulated and hypocellular in appearance, staining for IgG, as did the tubular basement membrane (TBM). On electron microscopy, there was evidence of granular electron dense material along the glomerular basement membrane (GBM) and TBM, which is a typical finding of MIDD (Figures 1, 2). We were able to demonstrate clearly IgG heavy chain, with no demonstration of LC restriction on direct immunofluorescence (Figures 3, 4, 5). In summary, the findings were consistent with a rare form of heavy chain only monoclonal immunoglobulin deposition disease (HCDD). 

Following this, a bone marrow biopsy and trephine showed low volume plasma cell infiltrate (PC 3-4%) with kappa predominance. The combination of low-level plasma cells on bone marrow aspirate and trephine biopsy (BMAT), absence of any other multiple myeloma defining criteria and evidence of a monoclonal immunoglobulin-associated renal injury is consistent with a diagnosis of MGRS.

Our final diagnosis is HCDD in the context of MGRS.

Initially, he responded well to diuretics, angiotensin-converting enzyme (ACE) inhibition with improvement of the nephrotic state, preserved renal function and proteinuria of around 1 gr/d. During follow-up over six months, he developed worsening oedema, worsening renal function, proteinuria, hypoalbuminemia, rapidly increasing kappa light chains and SFLC ratio, and evidence of kappa light chain paraprotein on SPEP and immunofixation, fulfilling SLiM myeloma criteria based on the International Myeloma Working Group diagnostic criteria. The patient developed IgGk with an SFLC ratio > 100. 

Clone-directed chemotherapy with bortezomib, cyclophosphamide and dexamethasone was initiated. He responded well to this treatment with normalising SFLC ratio and negative SPEP. Clinically, he remained well with no evidence of fluid overload or severe side effects from chemotherapy. Nephrotic syndrome and acute kidney injury resolved.

## Discussion

MIDD is a complication of plasma cell disorders, defined by abnormal deposits of monoclonal immunoglobulins along basement membranes, with negative Congo-red staining and absence of fibrillary, crystalline or microtubular appearance on electron microscopy, thus excluding amyloidosis [2,3]. MIDD can be further categorised depending on the type of deposit; these include light chain deposition disease (LCDD), HCDD, and light and heavy chain deposit disease (LHCDD), of which LCDD is the most common.

In patients with MIDD, serum creatinine is a strong, and often the only, predictor of renal outcome [2,3], highlighting the importance of early detection in the prognosis but these studies include mainly LCDD, and only a minority with HCDD and LHCDD. A recent HCDD case series showed that initial serum Cr did not predict renal outcomes, and instead chemotherapy was the significant predictor [4]. This is likely due to early diagnosis, with baseline average estimated glomerular filtration rate (eGFR) of 50.4 ml/min/1.73m^2, responding well to therapy. This study provides further support that earlier initiation of treatment improves outcomes. 

MIDD usually presents in males over 50 with a moderate to severe degree of proteinuria (frequently nephrotic range in HCDD), hypertension and renal insufficiency, with a minority needing dialysis at the time of biopsy [2,5]. This is often coupled with dysproteinaemia and underlying haematological disease, commonly MGRS or multiple myeloma (Table 1). Table 1 summarizes the key clinical findings of all the major studies published to date on MIDD. Across these cohorts, HCDD remained rare, 0-16% of all MIDD cases. Hypertension, impaired renal function and severe proteinuria (often with nephrotic syndrome) are the main clinical features. It is important to clarify that these case series include biopsy-proven MIDDs and data whether multiple myeloma or MGRS findings were concurrent or follow-up findings are missing. 

**Table 1:** Major case series with HCDD CLL, Chromic Leukemia; CN, cast nephropathy; eGFR, estimated Glomerular Filtration Rate, HCDD, Heavy Chain Deposition Disease; HTN, Hypertension; LCDD, Light Chain Deposition Disease; LHCDD, Light and Heavy Chain Deposition Disease; MGUS, Monoclonal Gammopathy of Undetermined Significance; MGRS, Monoclonal Gammopathy of Renal Significance; NR, Not Reported; sCr, serum creatinine; SFLC, Serum Free Light Chain, SIFE, Serum Immunofixation; SPEP, Serum Protein Electrophoresis; UPEP, Urine Protein Electrophoresis; UIFE, Urine protein immunofixation; WM, Waldnenstrom Macroglobulinaemia.

Author, year	Nasr et al., 2012 [2]	Pozzi et al., 2003 [12]	Kourellis et al., 2016 [5]	D.C Ziogas et al., 2016 [6]	Sayed et al., 2015 [11]	Joly et al., 2019 [8]
No of patients	64	63	88	18	53	255
Mean age (range/SEM)	56 (22-83)	58 +/- 14.2	Median 56 (22-83)	66 (46-85)	Median 56 (29-78)	64 (53-75)
Male/Female	42/22	40/23	58/30	8/10	37/16	133/122
Histopathology, n						
LCDD	51	63	74	14	53	63
HCDD	7	0	7	3	0	23
LHCDD	6	0	7	1	0	57
LCDD +CD	0	0	0	0	0	67
Renal Characteristics						
HTN, %	83	NR	NR	89	94	55
Median sCr, mg/dl (range)	Mean 3.9 (0.9-15)	3.8	3 (0.9-15)	2.95 (0.7-13.2)	NR	269umol/l (169-471)
Median eGFR, ml/min (range)	NR	NR	22 (2.5-83)	30.7 (5.2-99.3)	Mean 27 (0-79)	24.3 (11.9-42.9)
Dialysis at biopsy, %	16	NR	18	NR	17	23
Median 24h protein, g (range)	Mean 4.1	2.7	2.5 (5.4-17.6)	3.3 (0.4-4.6)	NR	3.3 (2.9-4)
Nephrotic range proteinuria, %	39 (>3gr)	40 (>3.5gr)	42 (>3gr)	50 (>3.5gr)	53 (>3gr)	NR
Median serum albumin, g/dl (range)	Mean 3.5	NR	3.5 (1.7-4.6)	3.3 (2.4-4.6)	NR	3.3 (2.9-4)
Odema, %	60	NR	NR	NR	NR	NR
Nephrotic syndrome, %	23	NR	NR	NR	22	22
Microscopic haematuria, %	62	NR	NR	NR	90	58
Haematological Characteristics, %						
Positive SPEP/SIFE	73	76	64	78	43	NR
Positive UPEP/UIFE	81	0.9	68	67	NR	NR
Abnormal SFLC ratio	100	NR	99	100	100	100
Light chain type, kappa/lambda	81/19	68/32	83/17	64/36	81/19	81/19
Monoclonal Gammopahty, %						
None	22	NR	0	NR	NR	10
IgG	31	NR	58	NR	NR	39
IgA	13	NR	27	NR	NR	14
IgM	0	NR	14	NR	NR	6
Other	0	NR	1	NR	NR	30
Underlying pathlology/haematological diagnosis, %						
Multiple Myeloma	59	65	21	0	NR	34
MGUS/MGRS	NR	32	42	NR	NR	64
WM	NR	0	2	NR	NR	1
Smouldering	NR	0	36	NR	NR	NR
CLL	NR	3	0	NR	2	0.4
B Cell Lymphoma	NR	0	0	NR	NR	0.8

Heavy proteinuria may be due to complement activation by heavy chains [2], which may cause stimulation of mesangial cells leading to glomerular injury and thus greater proteinuria [6,7]. Supporting this, some studies have shown that patients with the disease can present with hypocomplementemia [8,9]. There has also been evidence of deletion of the CH1 domain in cases of HCDD [8,10], which although not fundamental for diagnosis, could be an important part of the pathogenesis.

Extra-renal manifestations of MIDD can also be seen [2,8], especially cardiac involvement, which confers a poor prognosis. As findings can often resemble cardiac amyloid, confidently excluding a diagnosis of amyloidosis may prove challenging prior to biopsy. 

Dysproteinaemia is very common in MIDD, with SFLC ratio being a highly sensitive marker, found in 100% of the patients in most studies thus far [2,5,6,8,11,12]. Paraprotein on urine or serum protein electrophoresis is not always seen. In our case, the only relevant finding prior to kidney biopsy was a modest rise in SFLC ratio, although this can often be normal with a degree of renal impairment. Interestingly, Lin et al. [3] showed that renal biopsy diagnosis preceded any evidence of dysproteinaemia in the majority of patients with MIDD. This lack of biochemical evidence in early disease may pose some difficulty regarding the decision to perform a renal biopsy, specifically for patients with established diabetes, thus delaying diagnosis and possibly long-term outcomes.

The optimal approach to managing MIDD in the context of MGRS is not known due to a lack of prospective, controlled studies. The current data are only from retrospective cohort studies but suggest that the depth of haematological response is important for renal and overall survival, even in MGRS [8,9].

Our patient was under active surveillance at the time of diagnosis, but eventually needed active treatment following evidence of clinical and biochemical deterioration. This further supports the need for early renal biopsy and accurate diagnosis. We could consider whether there is a place for pre-emptive treatment of MIDD if progression of disease may be inevitable without it. However, the decision to start chemotherapy in an otherwise clinically improving patient, following critical illness would need careful risk/benefit analysis, reiterating the importance of correlating histopathological and clinical findings and close follow-up, using a collaborative approach.

## Conclusions

In conclusion, in patients with unexplained proteinuria, especially when nephrotic-range or progressive, a renal biopsy should be strongly considered, as timely diagnosis and clone-directed therapy can prevent progression to ESKD. Biomarkers, such as, SFLC can guide decision to escalate from surveillance to therapy. Close collaboration between nephrologists, histopathologists and haematologists is needed in patients with monoclonal gammopathies of renal significance for prompt diagnosis and improved patients' outcomes.
